# Occurrence of an infectious complication may be a predictor of venous thromboembolism after surgery for colorectal cancer

**DOI:** 10.1016/j.rpth.2025.102886

**Published:** 2025-05-17

**Authors:** Stijn van Cruchten, Edgar M. Wong–Lun-Hing, Michel M.P.J. Reijnen, Marnix A.J. de Roos

**Affiliations:** 1 Department of Gastrointenstinal and Oncological Surgery, Rijnstate Hospital, Arnhem, the Netherlands; 2 Department of Vascular Surgery, Rijnstate Hospital, Arnhem, the Netherlands; 3 Multi-Modality Medical Imaging Group, TechMed Centre, Universteit Twente, Enschede, the Netherlands

**Keywords:** venous thromboembolism, surgical complications, colorectal neoplasms, surgical oncology, thrombosis

## Abstract

**Background:**

Venous thromboembolism (VTE) is a rare complication after colorectal cancer surgery, but may have a devastating outcome. The goal of this study was to report the incidence of VTE in our practice and identify predictors of VTE after colorectal resection for cancer.

**Methods:**

This was a single-center retrospective cohort analysis. We used the hospital-specific Dutch Colorectal Audit database to identify patients that underwent oncologic colorectal resection between 2015 and 2022 and subsequently developed a VTE. Patients who used therapeutic anticoagulants postoperatively due to pre-existing conditions were excluded. During the study period, VTE prophylaxis was applied according to the local protocol. Patient characteristics and postoperative data were extracted from the patient records.

**Results:**

Overall, 1261 patients were included, of which 13 patients developed VTE (1.0%). All cases involved pulmonary embolism. One patient (7.7%) had a simultaneous deep venous thrombosis. There were no deaths due to VTE. The incidence of other complications was significantly higher in patients with VTE (84.6% vs 28.5%; *P* ≤ .001). Multivariable logistic regression analysis indicated that the occurrence of an infectious complication was an independent predictor of VTE (odds ratio, 7.95; 95% CI, 2.20-28.69). Other variables that have previously been connected to the occurrence of VTE have been analyzed, but no other independent predictors were identified.

**Conclusion:**

An infectious complication may be an independent predictor of the development of VTE. The necessity of prolonged prophylaxis after oncologic colorectal resections remains unclear.

## Essentials

• Venous thromboembolism (VTE) is a rare but severe complication after colorectal surgery.
• Patients with and without a VTE after colorectal cancer surgery were compared.
• In the VTE group, other complications occurred significantly more often.
• In this cohort, an infectious complication was an independent predictor of VTE.

## 1 Introduction

Venous thromboembolism (VTE) is a rare but potentially severe complication after surgical treatment of colorectal carcinoma [1]. In the Dutch guideline, the type of surgical intervention and patient-related risk factors are considered to be decisive factors in the decision to prescribe prophylaxis. The guideline advices to consider prolonged pharmacologic prophylaxis in case of certain patient-specific risk factors [2]. The Enhanced Recovery After Surgery (ERAS) Society guideline for perioperative care in elective colorectal surgery, however, claims that all patients should receive pharmacologic prophylaxis for 28 days after major colorectal surgery [3]. The Dutch guideline is based on available literature on the subject and contains agreements and advice regarding VTE prophylaxis [4]. The guideline leaves space for interpretation, and therefore, differences in daily practice may exist across hospitals in the Netherlands. Centers may apply high-dose and prolonged VTE prophylaxis after all oncologic colorectal resections: low-molecular-weight heparin (LMWH) until 4 to 6 weeks postoperatively, whereas other hospitals may never provide prolonged prophylaxis after discharge or only in the presence of additional risk factors. Different considerations affecting this choice are a potentially not only higher risk of bleeding but also higher costs of prophylaxis due to the prolonged prescription of LMWH and a possible need for care and support at home. The goal of this study was to analyze the occurrence of VTE in a single-center cohort comprising patients who underwent colorectal surgery for cancer, to assess the presence of known risk factors of VTE in this cohort, and to investigate the influence of additional complications on the occurrence of VTE.

## 2 Methods

### 2.1 Study design

For this retrospective cohort analysis, data were collected from the Dutch Colorectal Audit (DCRA) database, a nationwide quality improvement audit initiated in 2009 [5]. The DCRA is a disease-specific audit and contains a wide range of variables with specific information regarding patient and tumor characteristics, diagnostics, treatment, complications, and mortality. Patients are excluded from this database in case of an endoscopic resection and/or in case of a recurrence colorectal carcinoma. For this analysis, data from the DCRA of all consecutive patients who had undergone a surgical resection for primary colorectal carcinoma at 1 single teaching hospital between 2015 and 2022 were used. The database was manually screened to identify all eligible patients. Both patients with and without an occurring VTE were included. Patients using therapeutic anticoagulants due to pre-existing conditions were excluded from the study. The 90-day postoperative morbidity is among the variables included in the DCRA database. Moreover, several studies claim a heightened postoperative VTE up to 12 weeks postoperatively [6,7]. This is why a VTE was defined as a deep venous thrombosis (DVT) or a pulmonary embolism (PE) within 12 weeks after surgery.

For this report, we adhered to the Strengthening the Reporting of Observation Studies in Epidemiology (STROBE) guidelines [8]. Retrospective research of patients’ files is not in the scope of Dutch law for human research; therefore, investigational review board approval was not required, but a waiver and local approval was obtained (Lokale Haalbaarheidscommissie (LHC) registration number 2023-2325). As a consequence, informed consent of the patients was not obtained. Electronic hospital records were checked to ensure patients had no objection for the use of data in scientific research. Patients’ data were pseudonymized. This study was preregistered in the institutional registry of Rijnstate hospital and approved by the ethics committee of the hospital.

### 2.2 Current VTE prevention protocol and daily practice

The local protocol considers an oncologic colorectal resection as a procedure with a high thrombotic risk. The protocol recommends postoperative prophylactic usage of nadroparin until adequate mobilization or discharge, and adjustments in the applied dose should be made according to patient’s weight. In addition, a prolonged usage of up to 4 weeks postoperatively should be considered in patients with 1 or more following risk factors: an age of >75 years, a body mass index of >30 kg/m^2^, a cerebrovascular accident in the month before surgery, a VTE in patient history, and existing hereditary coagulation disorders [4]. The recommendations in the local protocol are in line with those in the Dutch guideline.

### 2.3 Data collection

The primary variable of interest was the occurrence of VTE. Additional variables with known clinical relevance for the risk of occurrence of VTE were collected: all VTE risk factors mentioned in the Dutch guideline and additional factors after literature review (gender, tumor stage, infectious complications, American Society of Anesthesiologists [ASA] Physical Status Classification System) [9]. Gender refers to the classification of individuals as male or female based on information recorded in the electronic patient records at the time of registration. This classification typically reflects biological attributes but may not capture gender identity.

After patient selection the following variables were either extracted from the DCRA database or manually extracted from the health care information system (Hix version 6.3; Chipsoft): age, gender, body mass index, ASA classification, patient history (VTE, cerebrovascular accident, and coagulation disorder), other complications (perioperative and postoperative within 90 days after surgery), and preoperative and postoperative usage of anticoagulants. Postoperative bleedings were graded as major bleeding, clinically revelant nonmajor bleeding and minor bleeding based on the International Society on Thrombosis and Haemostasis bleeding scale [10,11]. For the VTE group, data regarding type of surgical procedure, tumor characteristics, and adjuvant chemotherapy were also extracted. The Clavien–Dindo classification was used to rank severity of surgical complications. This classification consists of 5 grades: a grade 1 complication is a postoperative deviation of expected course without the requirement of additional treatment. Grade 2 are complications requiring treatment with medication. Grade 3 complications require radiologic, surgical, or endoscopic interventions. Grade 4 complications are severe life-threatening complications requiring intensive care unit management, and a grade 5 complication means the patient has deceased as a consequence [12].

### 2.4 Statistical analysis

All statistical analyses were performed using IBM SPSS Statistics (version 22.0.0.2). Whenever applicable, data of the VTE group was compared with those of the non-VTE group. Continuous data were described as median (range) and categorical data as percentages. To identify differences in patient variables between the VTE and non-VTE group, the chi-squared was used for categorical data. When the observed cell count was 5 or lower, the Fisher exact test was used. The Mann–Whitney U-test was used to compare continuous data. Univariable and multivariable logistic regression analyses were conducted to identify possible VTE risk factors. Variables that were statistically significant in univariable analyses were included in the multivariable logistic regression analysis. A *P* value of <.05 was considered as statistically significant.

## 3 Results and Discussion

### 3.1 Patient selection and incidence

The initial group of patients from the DCRA consisted of 2352 patients. After excluding duplicate records (*n* = 25) and patients who were treated before 2015 (*n* = 914), 1413 patients were selected. The medical records of all these patients were manually reviewed by 2 independent authors (S.v.C., E.M.W.-L.-H.), resulting in the exclusion of an additional 24 patients who had either undergone endoscopic resection by a gastroenterologist or received no intervention. Another 128 patients using therapeutic anticoagulants were also excluded from the analysis. In 12 of 1261 patients, a VTE was registered as a postoperative complication. Based on the manual screening of medical records, 4 patients were excluded with incorrectly diagnosed postoperative VTE, and 5 patients were additionally identified with a VTE after surgery. Therefore, 13 of 1261 patients with a postoperative VTE were finally included in the analysis (Figure 1), leading to a postoperative VTE incidence of 1.0%. The baseline characteristics and surgical interventions are presented in Table 1 and were mostly comparable between the VTE and non-VTE groups. All 13 patients had PE, consisting of 10 segmental or subsegmental PE, 2 central PE, and 1 patient had both. All 13 patients had complaints of fever and increased C-reactive protein or malaise, and 9 had respiratory symptoms. PE was bilateral in 7 cases and unilateral in 6 patients. One patient had a simultaneous DVT, with thrombus in the external iliac vein, common and superficial femoral veins, popliteal vein, anterior and posterior tibial veins, and peroneal vein. All VTEs were diagnosed by computed tomography. DVT was confirmed by ultrasound duplex. The median time between surgery and the occurring VTE was 7 days (IQR, 4-16 days). No patients received postoperative chemotherapy before the VTE occurred.

**Figure:**
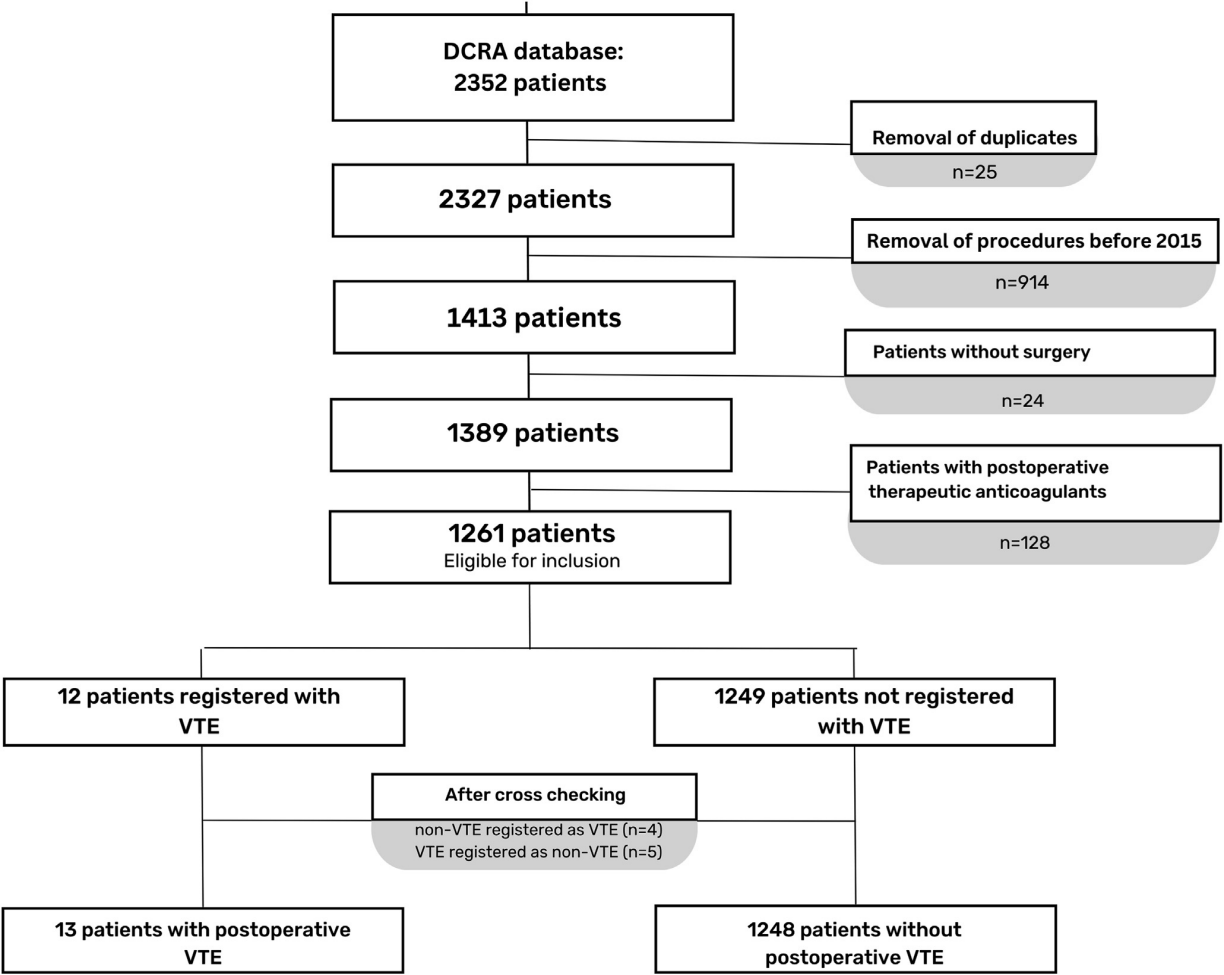
Flow chart of patient selection. DCRA, Dutch Colorectal Audit; VTE, venous thromboembolism.

**Table 1:** Baseline characteristics and surgical interventions of the cohort.

Characteristics	VTE group (*n* = 13)	Non-VTE group (*n* = 1248)	*P*
Male	8 (61.5)	716 (57.4)	1.000
Age (y), median (IQR)	71.0 (63.5-75.5)	69.0 (61.0-75.0)	.601
Body mass index (kg/m^2^), median (IQR)	27.4 (24.1-31.5)	26.1 (23.6-29.0)	.226
ASA classification			.641
1	2 (15.4)	173 (13.9)	
2	6 (46.1)	729 (58.4)	
3	5 (38.5)	310 (24.8)	
4	0 (0.0)	36 (2.9)	
T-stage			.929
1	1 (7.7)	129 (10.3)	
2	6 (46.1)	392 (31.4)	
3	5 (38.5)	653 (52.3)	
4	1 (7.7)	74 (5.9)	
N-stage			.627
0	8 (61.5)	803 (64.3)	
1	5 (38.5)	338 (27.1)	
2	0 (0.0)	107 (8.6)	
M-stage			1.000
0	13 (100.0)	1192 (95.5)	
1	0 (0.0)	56 (4.5)	
Interventions			.352
Hemicolectomy, right	6 (46.1)	433 (34.7)	
Sigmoid resection	1 (7.7)	402 (32.2)	
Low anterior resection	2 (15.4)	202 (16.2)	
Hemicolectomy, left	2 (15.4)	115 (9.2)	
Other	2 (15.4)	96 (7.7)	
Laparoscopy of laparotomy			.258
Laparoscopy	10 (76.9)	1072 (85.9)	
Laparotomy	3 (23.1)	127 (10.2)	
TEM	0 (0.0)	49 (3.9)	
Anastomosis or stoma			.692
Anastomosis	11 (84.6)	1064 (85.3)	
Primary stoma	2 (15.4)	136 (10.9)	
TEM	0 (0.0)	48 (3.8)	

Values are *n* (%) unless specified.

VTE, venous thromboembolism; ASA, American Society of Anesthesiologists; T-stadium, tumor stage; N-stadium, node stage; M-stadium, metastasis stage; TEM, transanal endoscopic microsurgery.

The VTE incidence is lower than previously described in literature. In 2021, Lewis–Lloyd et al. [13] published a meta-analysis on the incidence of symptomatic VTE after oncologic colorectal surgery. Only randomized studies and population-based cohort studies were included. Their pooled analysis of 11 unique studies reported a mean incidence of 1.92% after 1 month [13]. A review by Emoto et al. [14] in 2019 presented incidences from large retrospective databases, varying between 1.15% and 2.47%. In this review, both studies using a 30-day postoperative VTE rate and studies investigating a 90-day VTE rate were included [14]. Recent publications found a VTE incidence of 1.5% (no postoperative period mentioned) [15] and 2.5% (up until 1 month postoperatively) [16], with an asymptomatic VTE incidence of 11.2% in the latter prospective study. The observed relatively low incidence of VTE in this study may be of a multifactorial nature. First, the ERAS protocol for colorectal surgery has been introduced and implemented and is part of a daily routing surgical care in our hospital [3]. This entails that directly postoperative early mobilization is strictly promoted and supported by nurses and a physical therapist. It is extensively known that mobilization diminishes the risk of VTE [[17], [18], [19]]. Moreover, the routine blood testing (C-reactive protein) on postoperative day 2 and 3 within the ERAS protocol facilitates earlier detection of complications or deviation from normal recovery. Most of the studies included in aforementioned reviews date from an earlier period. There may have been less compliance with the ERAS principles. Second, there has been an increase in the use of minimally invasive treatment methods. This also diminishes the risk of VTE. Part of the difference in incidence may be explained by underreporting. In patients with no significant clinical symptoms or with a likely different diagnosis for their complaints (eg, pneumonia in case of respiratory complaints), there has been no subsequent imaging to diagnose a VTE. Asymptomatic VTEs were not considered in the aforementioned studies and, therefore, do not explain the difference in VTE incidence observed in this study.

### 3.2 VTE group versus non-VTE group

In the VTE group, 84.6% of the patients had at least 1 other complication than VTE, compared with only 28.5% in the non-VTE group (*P* < .001). Both the rate of Clavien–Dindo complications of grades 1 to 2 (VTE 53.8% vs non-VTE 19.2% *P* = .006) and the rate of complications of grades 3 to 5 (VTE 30.8% non-VTE 9.5% *P* = .030) were significantly higher in the VTE group. Univariable and multivariable logistic regression analysis were conducted for risk factors of VTE. The results are depicted in Table 2. Significant morbidity within 90 days after surgery, defined as the occurrence of a complication classified as Clavien–Dindo grade 3 or higher (odds ratio [OR], 4.26; 95% CI, 1.29-14.03) and the occurrence of infectious complications (OR, 8.38; 95% CI, 2.72-25.89) were associated with a higher risk of VTE in the univariable analysis. These 2 factors were included in the multivariable analysis. The occurrence of an infectious complication remained a significant factor after multivariable analysis for VTE in this cohort (OR, 7.95; 95% CI, 2.20-28.69). The occurrence of serious complications (Clavien–Dindo grade of 3 or higher) was not independently associated with the occurrence of VTE (OR, 1.13; 95% CI, 0.29-4.42).

**Table 2:** Univariable and multivariable analysis of risk factors for VTE.

Variable	VTE group (*n* = 13)	Non-VTE group (*n* = 1248)	*P*, univariable analysis	Odds ratio (95% CI)	*P*, multivariable analysis	Odds ratio (95% CI)
Male/female	8/5	716/532	1.000	0.84 (0.27-2.59)		
ASA classification ≥3 (yes/no)	5/8	346/902	.367	1.63 (0.53-5.02)		
Age >75 y (y/n)	3/10	293/955	1.000	0.98 (0.27-3.58)		
Obesity (BMI >30 kg/m^2^) (yes/no)	5/8	237/1011	.085	2.67 (0.86-8.22)		
VTE in patient history (yes/no)	1/12	30/1218	.278	3.38 (0.43-26.86)		
Hereditary coagulation disorders (yes/no)	0/13	3/1245	1.000	—		
Recent CVA (<1 mo) (yes/no)	0/13	2/1246	1.000	—		
Infectious complications (within 90 d after surgery) (yes/no)	8/5	200/1048	**<.001**	**8.38 (2.72-25.89)**	**.002**	**7.95 (2.20-28.69)**
Significant morbidity within 90 d after surgery (≥Clavien Dindo 3) (yes/no)	4/9	118/1130	**.017**	4.26 (1.29-14.03)	.860	1.13 (0.29-4.42)
Tumor ≥ T3-T4 (yes/no)	6/7	727/521	.384	0.61 (0.21-1.84)		

Boldface values are statistically significant.

ASA, American Society of Anesthesiologists; BMI, body mass index; CVA, cerebrovascular accident; VTE, venous thromboembolism.

The occurrence of an infectious complication postoperatively may be an independent risk factor for the occurrence of a postoperative VTE. This is in line with a risk assessment model published by Iannuzzi et al. [20] in 2013, based on a retrospective analysis. They found a major postoperative complication to be an independent predictor of postdischarge VTE. The occurrence of a postoperative infection was not analyzed as an independent risk factor [20]. In addition, Gangireddy et al. [21] reported postoperative infectious complications as a predicting factor associated with VTE. This may be explained by a state of increased coagulability that is caused by a combination of systemic inflammation, repeated intervention, and a prolonged period of reduced mobility [20,22,23].

In total, 511 patients (40.9%) in the non-VTE group had an at least 1 risk factor that was associated with the occurrence of postoperative VTE, compared with 5 patients (53.8%) of the patients in the VTE group. No significant differences in presence of risk factors were observed between both groups (VTE vs non-VTE): 1 risk factor, 46.2% vs 37.0%; 2 risk factors, 7.7% vs 3.8%; and 3 risk factors, 0.0% vs 0.3% (*P* = .888). These risk factors have been previously studied. A recent prospective cohort study by Wei et al. [16] showed that high age (>70 years) was an independent risk factor for postoperative VTE. Other studies that have analyzed risk factors for VTE after all types of surgery found positive predictors for VTE to be age, obesity, malignancy, ASA classification, increased operative time and postsurgical stay, acute renal insufficiency, postoperative transfusion, perioperative myocardial infarction, and pneumonia [20,21]. Moreover, a history of varicose veins in the lower extremities, cardiac insufficiency, female gender, preoperative bowel obstruction, preoperative bloody stool, and anesthesia time of >180 minutess were associated with VTE [16].

### 3.3 Prophylaxis and VTE treatment

Overall, all patients received VTE prophylaxis; 1257 patients (99.7%) used prophylaxis until discharge, and 3 (0.2%) patients, all in the non-VTE group, received prolonged prophylaxis. One patient (0.1%) in the VTE group received prophylaxis, but this was terminated during the hospital stay. No explanation for these protocol deviations were documented. The median duration of prophylaxis was 5 days (IQR, 4-8 days). Seven patients were still hospitalized and used nadroparin in low prophylactic dose (53.8%) when the VTE occurred.

In the VTE group, 38.5% of the patients used an antiplatelet agent (*n* = 5), compared with 16.6% of the non-VTE group (*n* = 207; *P* = .052). Two of 5 patients who already used antiplatelet agents preoperatively (40.0%) had not fully resumed their own medication when the VTE occurred (at day 3 and day 7, postoperatively).

The VTE treatment consisted in 3 patients (23.1%) of nadroparin injections for 3 months, in 3 patients (23.1%) a short period of nadroparin injections was followed by acenocoumarol for 6 months (*n* = 1) or 3 months (*n* = 2). Seven patients (53.8%) were treated with nadroparin, followed shortly by rivaroxaban. Of these, 3 patients used rivaroxaban for 3 months and 1 for 1 year, and 3 patients were required to use it for the rest of their lives.

As far as the medical records showed, all patients recovered completely after drug therapy. One patient in the VTE group died within 90 days after the VTE as a result of cancer recurrence.

Overall, 14 postoperative hemorrhages (1.1%) occurred in this cohort, all in non-VTE group. Nine patients (64.3%) were observed during initial admission, and 5 (35.7%) patients were readmitted. Two patients (14.3%) had a major bleeding at the surgical site and required laparoscopy. Twelve patients had a clinically revelant nonmajor bleeding (85.7%): in 10 (83.3%) patients located at the surgical site and in 2 (16.7%) patients in the abdominal wall. In 6 patients (50.0%), observation and temporary stopping LMWH or antiplatelet agents was sufficient, 3 patients (25.0%) received tranexamic acid to stop the bleeding, 2 patients (16.7%) needed blood transfusions, and 1 (8.3%) patient had rectal blood loss stopped after inserting a rectal tampon. All patients with a postoperative bleeding were receiving VTE prophylaxis at that time.

Both the Dutch guideline and the local protocol regarding VTE prophylaxis define the same risk factors for postoperative VTE and recommend consideration of prolonged prophylaxis in the presence of one of these risk factors [2,4]. Nonetheless, in both the VTE and non-VTE groups, prolonged VTE prophylaxis was rarely prescribed. Considering the low incidence of VTE observed in this study, this finding suggests that the use of prolonged VTE prophylaxis could be approached with greater caution. On the contrary, existing literature indicates that the bleeding risk associated with prophylactic nadroparin is deemed acceptable and does not increase the incidence of major bleeding [24]. Unfortunately, the benefit of prolonged prophylaxis in a subset of high-risk patients could not be determined from this study and should be further investigated in future research.

Although the number of patients is substantial for this single-center study with low reported incidence of VTE, the overall group size can be considered a limitation for the study. The retrospective study design is another limitation. Not all risk factors for VTE known from literature were available for analysis, which might have limited the assessment of potentially relevant predictors.

In conclusion, in this study, the incidence of VTE after surgery for colorectal cancer was relatively low. Postoperative infection may be an independent risk factor for VTE. These findings provide new insights into the occurrence of VTE in an era where minimally invasive surgical procedures and perioperative optimization have been rapidly evolving. Interestingly, well known and previously defined risk factors were not significantly associated with the occurrence of VTE. Whether prolonged VTE prophylaxis with nadroparin is beneficial in a subset of high-risk patients could not be determined from this study.
